# Depressive symptoms as risk factors for the onset of home hypertension: a prospective cohort study

**DOI:** 10.1038/s41440-024-01790-9

**Published:** 2024-07-10

**Authors:** Sayuri Tokioka, Naoki Nakaya, Rieko Hatanaka, Kumi Nakaya, Mana Kogure, Ippei Chiba, Kotaro Nochioka, Hirohito Metoki, Takahisa Murakami, Michihiro Satoh, Tomohiro Nakamura, Mami Ishikuro, Taku Obara, Yohei Hamanaka, Masatsugu Orui, Tomoko Kobayashi, Akira Uruno, Eiichi N. Kodama, Satoshi Nagaie, Soichi Ogishima, Yoko Izumi, Nobuo Fuse, Shinichi Kuriyama, Atsushi Hozawa

**Affiliations:** 1 Tohoku University Graduate School of Medicine, Sendai, Japan; 2 Tohoku Medical Megabank Organization, Tohoku University, Sendai, Japan; 3 Tohoku University Hospital, Tohoku University, Sendai, Japan; 4 Tohoku Medical and Pharmaceutical University, Sendai, Japan; 5 Kyoto Women’s University, Kyoto, Japan; 6 International Research Institute of Disaster Science, Tohoku University, Sendai, Japan

**Keywords:** Cohort study, Depressive symptoms, Home blood pressure, Hypertension

## Abstract

Depression is comorbid with somatic diseases; however, the relationship between depressive symptoms and hypertension (HT), a risk factor for cardiovascular events, remains unclear. Home blood pressure (BP) is more reproducible and accurately predictive of cardiovascular diseases than office BP. Therefore, we focused on home BP and investigated whether depressive symptoms contributed to the future onset of home HT. This prospective cohort study used data from the Tohoku Medical Megabank Community-Cohort Study (conducted in the Miyagi Prefecture, Japan) and included participants with home normotension (systolic blood pressure (SBP) < 135 mmHg and diastolic blood pressure (DBP) < 85 mmHg). Depressive symptoms were evaluated using the Center for Epidemiologic Studies Depression Scale-Japanese version at the baseline survey. In the secondary survey, approximately 4 years later, the onset of home HT was evaluated (SBP ≥ 135 mmHg or DBP ≥ 85 mmHg) and was compared in participants with and without depressive symptoms. Of the 3 082 (mean age: 54.2 years; females: 80.9%) participants, 729 (23.7%) had depressive symptoms at the baseline survey. During the 3.5-year follow-up, 124 (17.0%) and 388 (16.5%) participants with and without depressive symptoms, respectively, developed home HT. Multivariable adjusted odds ratios were 1.37 (95% confidence interval (CI): 1.02–1.84), 1.18 (95% CI: 0.86–1.61), and 1.66 (95% CI: 1.17–2.36) for home, morning, and evening HT, respectively. This relationship was consistent in the subgroup analyses according to age, sex, BP pattern, and drinking habit. Depressive symptoms increased the risk of new-onset home HT, particularly evening HT, among individuals with home normotension.

**Figure:**
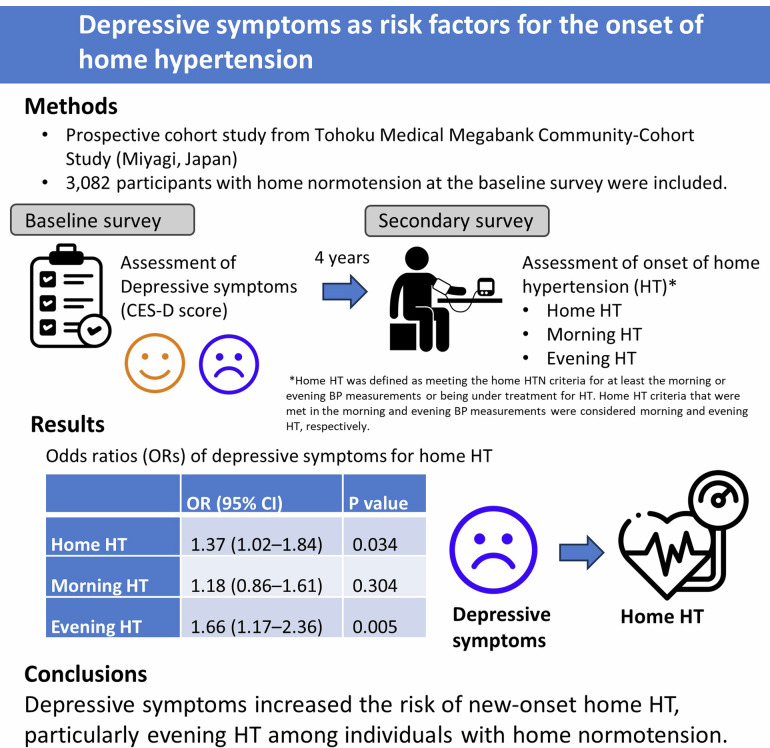
This prospective cohort study revealed that depressive symptoms are risk factors for new-onset home hypertension, particularly evening hypertension among individuals with home normotension. Assessing home blood pressure in individuals with depressive symptoms is important for the prevention of hypertension and concomitant cardiovascular diseases.

This prospective cohort study revealed that depressive symptoms are risk factors for new-onset home hypertension, particularly evening hypertension among individuals with home normotension. Assessing home blood pressure in individuals with depressive symptoms is important for the prevention of hypertension and concomitant cardiovascular diseases.

## Introduction

Depression is a common mental disorder, which is frequently comorbid with somatic diseases [1, 2]. It is reported that depression is a risk factor for cardiovascular diseases (CVD) and it has a poor prognosis [3–5]. This relationship is due to the fact that individuals with depressive symptoms often have CVD risk factors including obesity, unhealthy diet, inactivity, smoking, and poor medication adherence [1]. Additionally, mental stress causes autonomic nervous system (ANS) and adrenocortical hormone dysregulation [6, 7], which may result in CVD [1].

Hypertension (HT) is an important risk factor for CVD, and blood pressure (BP) is influenced by depression, anxiety, stress, and personality traits [8–11]. However, the relationship between depression and HT remains unclear [12]. Some studies have reported a positive association between depression and HT [13–15], whereas others have reported a negative association [16–19].

Most studies assessed only office BP, and this may be a reason for inconsistent results [13–19]. We previously reported the positive association between depressive symptoms and masked HT (MHT) in participants with normotension at a research center in a cross-sectional study [20]. The study suggested that it is important to assess home BP in individuals with depressive symptoms, for early diagnosis and management of HT [20]. Guidelines recommend prioritizing home BP in the treatment of HT, and MHT requires anti-hypertensives [21–24]. Additionally, home BP monitoring offers several advantages [25]. First, home BP is measured in a familiar environment. Second, home BP is more reproducible, and thus predicts CVD more accurately. Third, home BP is measured both in the morning and evening. Therefore, assessing home BP may be more beneficial for individuals with depressive symptoms.

However, the causal relationship between depressive symptoms and home hypertension remains unclear. Thus, this study aimed to investigate whether depressive symptoms increase the risk of new-onset home HT using home BP measurements to optimize the management of depressive symptoms for better BP control and prognosis. We hypothesized the possibility of a positive association between depressive symptoms and new-onset home HT. Additionally, we also assessed both BP measured at a research center (research BP) and at home to investigate whether research BP and home BP are differently affected by depressive symptoms.

Point of view

- **Clinical relevance**:
  This study showed depressive symptoms increased the risk of new-onset home hypertension among individuals with home normotension. It is suggested that monitoring home blood pressure in individuals with depressive symptoms may be useful for early diagnosis and management of hypertension.
- **Future direction**:
  It is unclear that monitoring home BP in individuals with depressive symptom is effective to improve their prognosis, and further study is warranted.
- **Consideration for the Asian population**:
  Approximately 150 million individuals are estimated to have depression in Asia. This study may contribute to improving prognosis of many individuals with depressive symptoms in Asia by promoting home BP monitoring.

## Methods

### Study participants

This prospective cohort study used data from the Tohoku Medical Megabank Community-Based Cohort Study (TMM CommCohort Study), a prospective cohort study conducted in the Miyagi Prefecture, Japan [26]. The baseline survey was conducted between 2013 and 2016. Participants were recruited via 3 approaches: (i) a type 1 survey collected basic data, including blood and urine tests, questionnaire responses, and municipal health checkup data at the specific health checkup sites of the annual community health examination; (ii) a type 1 additional survey which comprised the same tests, measurement, and questionnaires as that in the type 1 survey, was conducted in places selected by the municipality and the TMM CommCohort Study on dates that differed from the specific health checkup dates; and (iii) a type 2 survey was conducted at the Community Support Center with physical examination, blood and urine tests, and detailed measurements. Approximately 4 years after the baseline survey, a secondary survey was conducted at the Community Support Center between June 2017 and March 2021, with similar examinations as that in the type 2 baseline survey [27].

The eligibility criteria were as follows: (a) participation in type 2 and secondary surveys of the TMM CommCohort Study; (b) having home BP measured on at least 3 days during both the baseline and secondary survey periods [28, 29]; (c) not having home HT, which was defined as meeting the home HT criteria (SBP ≥ 135 mmHg or DBP ≥ 85 mmHg) for at least the morning or evening BP measurements or undergoing treatment for HT at the time of the baseline survey; and (d) having undergone complete assessment using the Center for Epidemiologic Studies Depression Scale (CES-D)-Japanese version at the baseline survey.

**Fig. 1:**
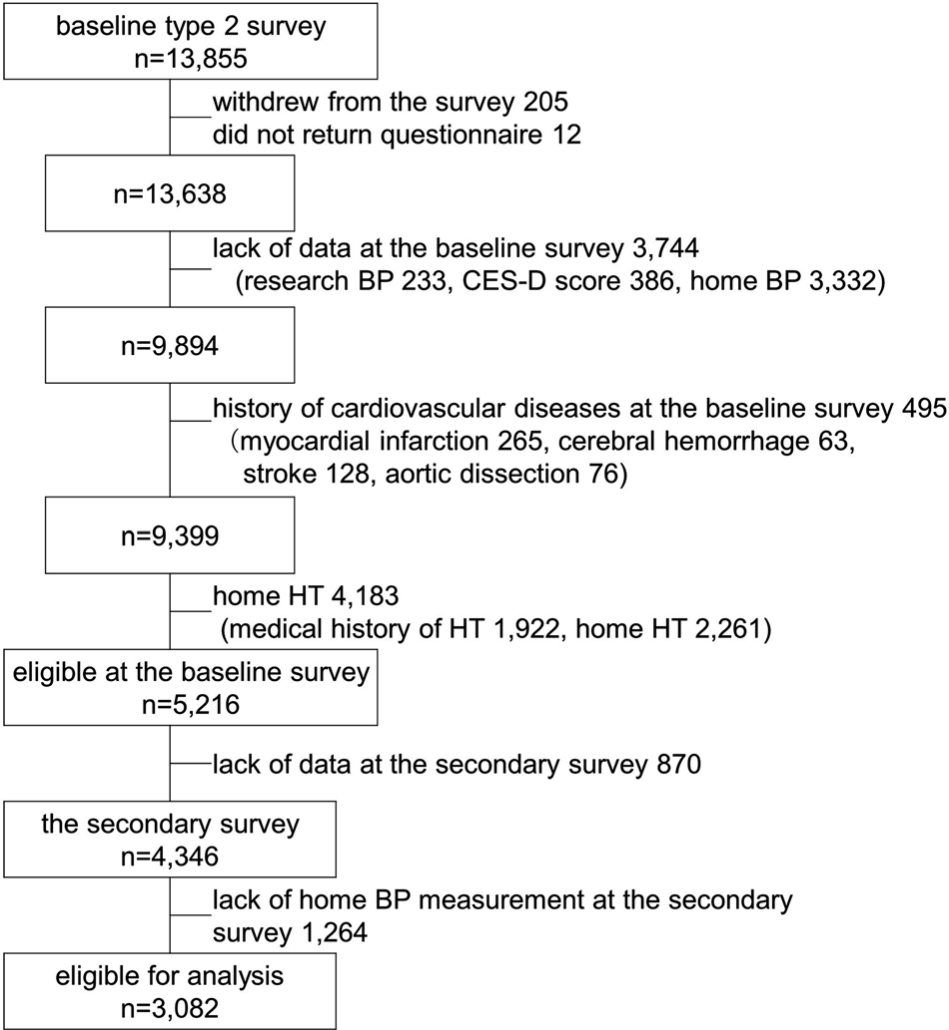
Selection of eligible participants from among the baseline and secondary survey data of the Tohoku Medical Megabank Community–Based Cohort study. BP Blood pressure, CES-D Center for Epidemiologic Studies Depression Scale, HT Hypertension

Participants who withdrew from the study by October 5, 2021 (*n* = 205); did not return self-report questionnaires (*n* = 12); had missing data on research or home BP measurements and CES-D (*n* = 344); had a medical history of CVD (*n* = 495); or had home or treated HT (*n* = 4183) were excluded from the baseline survey. Among the 5216 participants who satisfied the inclusion criteria at the baseline survey, those who did not participate in the secondary survey (*n* = 870) and those with missing home BP data (*n* = 1264) were excluded. Finally, 382 participants were included in the analysis (Fig. 1).

This study conformed to the Declaration of Helsinki guidelines and was approved by the Institutional Review Board of the Tohoku Medical Megabank Organization (Approval number: 2022-4-160). All the participants provided written informed consent.

### Data collection and measurements at the baseline survey

#### CES-D and definition of depressive symptoms

The CES-D self-report questionnaire was used to assess the depressive symptoms [30–32]. It comprises 20 items (16 positive statements and 4 negative statements), with a 0–3 ranking for each item. A cutoff score of 15/16 is widely used for depression screening in Japan [33]. In the present study, CES-D scores ≥ 16 were defined as depressive symptoms regardless of the use of anti-depressants.

#### Home BP

An electronic upper arm cuff device (HEM-7080IC; OMRON Corp., Kyoto, Japan) was used for home BP measurement. Participants measured their home BP and heart rate (HR) every morning and evening for 2 weeks in the baseline survey and for 10 days in the secondary survey. In accordance with Japanese guidelines, morning BP was measured while seated within 1 h of awakening and after 1–2 min of rest, before breakfast and medication intake [22]. Evening BP was measured 1–2 min after sitting before going to sleep. The respective mean of the morning and evening BP measurements, instead of the average of the morning and evening BP, were used in excluding participants and in the definition of the outcome. Average home BP, which was defined as the average of morning and evening BP, was also calculated and used in the model as a covariate. Moreover, changes in research BP and home BP between the baseline and the secondary surveys were also evaluated.

#### Research BP

The BP and HR measured at our Community Support Center were defined as research BP and HR, respectively [34]. After a rest period of 1–2 min, a trained nurse measured the BP twice in seated participants using an electronic upper arm cuff device (HEM-9000AI; OMRON Corp.) [34]. The mean of the two BP measurements was analyzed.

#### Physical examinations, laboratory data, and questionnaires

Physical examinations included measurements of height (AD-6400; A&D Co., Ltd., Tokyo, Japan), weight (InBody720; Biospace Co., Ltd., Seoul, Republic of Korea), and body mass index (BMI). The following blood test data were extracted: γ-glutamyl transpeptidase, hemoglobin A1c, low- and high-density lipoprotein cholesterol, triglycerides, and creatinine levels. Daily sodium intake was estimated using Tanaka’s method [35].

Lifestyle habits, including smoking, drinking, exercise, medical history, Athens Insomnia Scale (AIS) score, and educational background were obtained using self-report questionnaires. Smoking status was categorized as never, former, or current smokers. Drinking habit was defined as those who answered they drank regardless of the frequency and amount of alcohol consumption. Alcohol consumption per day was calculated based on drinking habits-related questionnaires (including the alcohol type, frequency, and amount) as follows:

(frequency of alcohol consumption per week ×amount of alcohol consumed on a single occasion)∕7.

The participants were asked the frequency and duration of their exercise performance according to exercise intensity. Regular exercise was defined as any type of exercise performed at least once a week. Regarding self-reported medical history, data on HT, diabetes mellitus (DM), dyslipidemia, stroke, heart failure, and myocardial infarction were collected. The AIS was used to screen for insomnia. It comprised eight items on sleep, with a 0–3 ranking for each item [36, 37]. A higher AIS score indicated more severe depressive symptoms. The AIS score was considered a continuous variable in the analysis. Educational status was categorized as less than high school, high school, more than high school, or others.

Additionally, the examination year and extent of house damage caused by the 2011 Great East Japan Earthquake were considered because of the possible post-traumatic physical and mental effects on the participants. The extent of house damage was categorized into 6 degrees (from “no damage” to “total damage”). The season at the examination time was included because it could influence BP and mood disorders [38, 39]. Winter was defined as the period from December to February, according to the mean temperature in the Miyagi Prefecture.

### Outcome

The main outcome was new-onset home HT. Home HT was defined as meeting the home HT criteria (SBP ≥ 135 mmHg or DBP ≥ 85 mmHg) for at least the morning or evening BP measurements or being under treatment for HT (treated HT) at the secondary survey. Home HT criteria that were met in the morning and evening BP measurements were considered morning and evening HT, respectively. Participants with treated HT were included in both morning and evening HT, despite their home BP values.

### Statistical analysis

Data were presented as mean ± SDs or medians (25th–75th percentile) for continuous variables with normal or skewed distributions, respectively, and as numbers (percentages) for categorical variables. The characteristics of participants with and without depressive symptoms were compared using the t-test for normal distribution (Student’s and Welch’s t-tests for 2 groups with similar and dissimilar variances, respectively), Mann–Whitney U-test for skewed distribution, and Chi-square test for categorical variables. Age- and sex-adjusted least squares (LS) means were calculated for BP and HR. To evaluate the association between depressive symptoms and the onset of home HT, multivariate logistic regression models were used to obtain the odds ratio (OR) and 95% confidence interval (CI). Complete case analysis was performed using 4 models: model 1, age and sex; model 2, model 1 plus research HR, BMI, dyslipidemia, DM, estimated glomerular filtration rate, smoking status, alcohol consumption, urinary sodium-to-potassium ratio, AIS score, and regular exercise; model 3, model 2 plus educational level, house damage, and examination in winter; and model 4, model 3 plus average SBP in the analysis of home HT, morning SBP in the analysis of morning HT, or evening SBP in the analysis of evening HT. All the data in the models were collected from the baseline survey.

Subgroup analysis was conducted according to age ( ≤ 56 and > 56 years), sex, BP pattern (normotension and white coat hypertension: WCHT), and drinking habit at the baseline survey. Normotension was defined as meeting the criteria of normotension at both research BP and home BP, and WCHT was defined as meeting the criteria of normotension at research BP and home HT at either morning or evening BP, or both. The cut-off values for age were median rounded off. The interaction of each category with depressive symptoms was analyzed by adding variables and multiplying depressive symptoms and categories into the model. Model 4 was used in the analysis, and sex in the subgroup analysis of sex and alcohol consumption in the subgroup analysis of drinking habit were removed from the model.

For sensitivity analysis, a multivariate analysis excluding participants with treated HT at the secondary survey was performed to evaluate the association between depressive symptoms and the onset of home HT.

*P*-values < 0.05 were considered statistically significant. All analyses were performed using R version 4.2.1 for Linux.

## Results

In total, 3 082 participants (mean age: 54.2 years; women: 80.9%) were included in the analysis (Fig. 1). Among these, 729 (23.7%) participants had depressive symptoms. Participants characteristics at the baseline survey are listed in Table 1. There were more women with depressive symptoms (88.6%). Participants with depressive symptoms were younger (age: 50.3 vs. 55.4 years, respectively) and had lower hemoglobin A1c, low-density lipoprotein cholesterol, and higher estimated glomerular filtration rate than those without depressive symptoms. Individuals with depressive symptoms consumed more alcohol and exercised less frequently. Current smokers were more among the participants with depressive symptoms (8.9%), and the majority in both groups were never smokers. Although the mean SBP on all occasions was lower in participants with depressive symptoms than in those without depressive symptoms at the baseline survey, the LS means of age- and sex-adjusted SBP did not reveal significant between-group differences (Supplemental Table S1).

**Table 1:** Health-related and socioeconomic characteristics of participants with home normotension at the baseline survey: 2013–2016

	Participants *n* = 3082	Non-depressive symptoms *n* = 2353	Depressive symptoms *n* = 729	*P*-value
Age (year), mean ± SD	54.2 ± 12.6	55.4 ± 12.3	50.3 ± 12.8	< 0.001
Sex (female), *n* (%)	2492 (80.9)	1846 (78.5)	646 (88.6)	< 0.001
BMI, mean ± SD	21.7 ± 3.0	21.7 ± 2.9	21.6 ± 3.3	0.157
**Blood pressure and heart rate**, mean ± SD				
Research SBP (mmHg)	120.3 ± 14.1	121.1 ± 14.2	117.9 ± 13.7	< 0.001
Research DBP (mmHg)	74.2 ± 9.0	74.4 ± 9.1	73.6 ± 8.9	0.025
Research HR (/min)	65.9 ± 9.2	65.7 ± 9.2	66.6 ± 9.0	0.024
Number of morning BP measurements	13.1 ± 2.6	13.2 ± 2.5	12.9 ± 2.8	0.024
Morning SBP (mmHg)	116.8 ± 10.0	117.1 ± 10.0	115.8 ± 9.8	0.003
Morning DBP (mmHg)	69.9 ± 7.1	70.0 ± 7.1	69.6 ± 6.9	0.184
Morning HR (/min)	67.0 ± 7.7	66.6 ± 7.6	68.4 ± 7.8	< 0.001
Number of evening BP measurements	12.7 ± 2.7	12.8 ± 2.7	12.5 ± 2.9	0.001
Evening SBP (mmHg)	114.1 ± 10.2	114.3 ± 10.1	113.3 ± 10.4	0.017
Evening DBP (mmHg)	66.9 ± 7.2	66.6 ± 7.6	66.8 ± 7.5	0.948
Evening HR (/min)	69.0 ± 7.6	68.8 ± 7.6	69.5 ± 7.6	0.018
Average home SBP (mmHg)	115.4 ± 9.5	115.7 ± 9.4	114.6 ± 9.6	0.004
Average home DBP (mmHg)	68.4 ± 6.8	68.4 ± 6.7	68.2 ± 6.9	0.467
**Blood and urine tests**				
HbA1c (%), mean ± SD	5.41 ± 0.37	5.43 ± 0.37	5.37 ± 0.38	< 0.001
LDL (mg/dL), mean ± SD	125.4 ± 30.5	126.2 ± 30.7	122.8 ± 29.8	0.010
HDL (mg/dL), mean ± SD	67.6 ± 16.3	67.3 ± 16.3	68.3 ± 16.3	0.169
TG (mg/dL), mean ± SD	88.7 ± 54.9	88.7 ± 54.2	88.6 ± 57.3	0.961
G-GTP (IU), mean ± SD	23.9 ± 23.4	24.3 ± 24.1	22.5 ± 21.0	0.061
Cr (mg/dL), mean ± SD	0.64 ± 0.13	0.65 ± 0.13	0.62 ± 0.12	< 0.001
eGFR(mL/min/1.73 m^2^), mean ± SD	82.3 ± 15.2	81.6 ± 14.8	84.7 ± 16.3	< 0.001
Urine Na/K ratio, mean ± SD	3.2 ± 0.68	3.2 ± 0.67	3.3 ± 0.70	0.433
NaCl intake*, mean ± SD	12.2 ± 2.7	12.3 ± 2.7	12.0 ± 2.7	0.005
**Medical history**				
Dyslipidemia, *n* (%)	383 (12.4)	310 (13.2)	73 (10.0)	0.028
Diabetes mellitus, *n* (%)	71 (2.3)	51 (2.2)	20 (2.7)	0.445
**Lifestyle**				
Drinking habit	1628 (52.8)	1287 (54.7)	341 (46.8)	< 0.001
Alcohol consumption (g/day), median (IQR)	0.66 (0.00–6.98)	0.91 (0.00–7.65)	0.00 (0.00–5.01)	0.006
Smoking status, *n* (%)				0.017
Never smoker	2158 (70.0)	1638 (69.6)	520 (71.3)	
Former smoker	698 (22.6)	556 (23.6)	142 (19.5)	
Current smoker	223 (7.2)	158 (6.7)	65 (8.9)	
Regular exercise, *n* (%)	2021 (65.3)	1609 (68.4)	403 (55.4)	< 0.001
CES-D	12.0 ± 6.9	9.0 ± 4.1	21.6 ± 5.0	< 0.001
AIS	3.7 ± 3.0	2.9 ± 2.3	6.0 ± 3.6	< 0.001
**Educational level**, *n* (%)				0.623
Less than high school	108 (3.5)	82 (3.5)	26 (3.6)	
High school	1408 (45.7)	1087 (46.2)	321 (44.0)	
More than high school	1545 (50.1)	1168 (49.6)	377 (51.7)	
Others	15 (0.5)	12 (0.5)	3 (0.4)	
**House damage in the Great East Japan Earthquake in 2011**, *n* (%)				< 0.001
Total damage	242 (7.9)	150 (6.4)	92 (12.6)	
Major damage	221 (7.2)	168 (7.1)	53 (7.2)	
Half-damaged	285 (9.2)	209 (8.9)	76 (10.4)	
Partial damage	1358 (44.0)	1070 (45.5)	388 (53.2)	
No damage	854 (27.7)	667 (28.3)	187 (25.7)	
Unaffected area	57 (1.8)	44 (1.9)	13 (1.8)	
**Examination date**, *n* (%)				
Winter	996 (32.3)	751 (31.9)	245 (33.6)	0.419
Year				0.881
2013	236 (7.7)	177 (7.5)	59 (8.1)	
2014	1851 (60.1)	1413 (60.1)	438 (60.1)	
2015	872 (28.3)	666 (28.3)	206 (28.3)	
2016	123 (4.0)	97 (4.1)	26 (3.6)	

*AIS* Athens Insomnia Scale, *BMI* Body mass index, *BP* Blood pressure, *CES-D* Center for Epidemiologic Studies Depression Scale, *Cr* Creatinine, *DBP* Diastolic blood pressure, *eGFR* estimated glomerular filtration rate, *G-GTP* Gamma-glutamyl transpeptidase, *HbA1c* Hemoglobin A1c, *HDL* High-density lipoprotein, *HR* Heart rate, *LDL* low-density lipoprotein, *SBP* Systolic blood pressure, *TC* Total cholesterol, *TG* Triglyceride. Average home SBP and DBP were defined as the average of morning and evening SBP and DPB, respectively

*NaCl and K intake estimated using Tanaka’s method

Table 2 presents the BP, HR, and outcomes of the secondary survey. In LS means and 95% CIs of age- and sex-adjusted SBP and DBP, morning and evening SBP and evening DBP were significantly higher in participants with depressive symptoms than in those without depressive symptoms, although no difference was observed in research BP. Change in research BP and morning BP was not significantly different between the two groups. The change in the evening BP was larger in the group with depressive symptoms than in the group without depressive symptoms (change in evening SBP: 1.6 in depressive symptoms vs. 0.6 in non-depressive symptoms; change in evening DBP: 1.3 in depressive symptoms vs. 0.6 in non-depressive symptoms). In total, 388 (16.5%), 347 (14.7%), and 205 (8.7%) participants without depressive symptoms and 124 (17.0%), 93 (12.8%), and 81 (11.1%) participants with depressive symptoms met the criteria of home, morning, and evening HT, respectively. Of these, 15 (2.1%) and 40 (1.7%) participants with and without depressive symptoms, respectively, answered that they were taking anti-hypertensive treatment in the secondary survey.

**Table 2:** Age- and sex-adjusted least square means of blood pressure and change in blood pressure between the baseline and the secondary surveys, and the prevalence of home hypertension at the secondary survey 2017–2020

	Non–depressive symptoms *n* = 2353	Depressive symptoms *n* = 729	*P*-value
**Blood pressure and heart rate**, age- and sex- adjusted least square means (95%CI)			
Research SBP (mmHg)	121.0 (120.0–122.0)	121.0 (120.0–122.0)	0.767
Research DBP (mmHg)	74.9 (74.5–75.4)	75.0 (74.3–75.8)	0.786
Research HR (/min)	66.7 (66.3–67.0)	67.7 (67.1–68.3)	0.002
Morning SBP (mmHg)	119.2 (118.7–119.8)	120.5 (119.5–121.5)	0.020
Morning DBP (mmHg)	72.2 (71.7–72.6)	72.8 (72.1–73.4)	0.090
Morning HR (/min)	66.4 (66.0–66.8)	67.2 (66.6–67.8)	0.013
Evening SBP (mmHg)	116.0 (115.0–117.0)	117.0 (116.0–118.0)	0.011
Evening DBP (mmHg)	68.2 (67.8–68.6)	69.2 (68.5–69.9)	0.006
Evening HR (/min)	69.0 (68.7–69.4)	69.5 (68.9–70.1)	0.185
Average home SBP (mmHg)	117.7 (117.1–118.3)	119.0 (118.0–119.9)	0.009
Average home DBP (mmHg)	70.2 (69.8–70.6)	71.0 (70.3–71.6)	0.020
**Change in blood pressure between the baseline and secondary surveys, age- and sex- adjusted least square means (95% CI)**			
Research SBP (mmHg)	−0.7 (−1.3 to −0.1)	−0.1 (−1.0 to 0.9)	0.215
Research DBP (mmHg)	−0.3 (−0.7 to 0.03)	−0.1 (−0.7 to 0.5)	0.508
Morning SBP (mmHg)	1.3 (0.8–1.7)	2.0 (1.3–2.8)	0.063
Morning DBP (mmHg)	1.2 (0.9–1.5)	1.6 (1.1–2.1)	0.105
Evening SBP (mmHg)	0.6 (0.2–1.1)	1.6 (0.8–2.4)	0.017
Evening DBP (mmHg)	0.6 (0.3–1.0)	1.3 (0.8–1.9)	0.015
**Outcome** (not adjusted), n (%)			
Home HT	388 (16.5)	124 (17.0)	0.804
Morning HT	347 (14.7)	93 (12.8)	0.207
Evening HT	205 (8.7)	81 (11.1)	0.066
Under treatment for HT	40 (1.7)	15 (2.1)	0.640

*DBP* Diastolic blood pressure, *HR* Heart rate, *HT* Hypertension, *SBP* Systolic blood pressure

Average home SBP and DBP defined as average of morning and evening SBP and DPB, respectively

Morning HT, meeting home HT criteria (home HT on home BP measurement or under treatment for HT at the secondary survey) in the morning measurement; evening HT, meeting home HT criteria in the evening measurement; and home HT, either or both morning or evening HT

Multivariate logistic regression analysis revealed a positive association between depressive symptoms and home HT, although no significant association was observed in the crude model (Table 3). The ORs for home and evening HT in model 4 were 1.37 (95% CI): 1.02–1.84) and 1.66 (95% CI: 1.17–2.36), respectively. Although no significant association was found between depressive symptoms and morning HT (OR: 1.18, 95% CI: 0.86–1.61), the LS means of morning SBP was significantly high in participants with depressive symptoms.

**Table 3:** Odds ratios and 95% CIs for home hypertension in participants with depressive symptoms

	Home HT		Morning HT		Evening HT	
	OR (95%CI)	*P*-value	OR (95%CI)	*P*-value	OR (95%CI)	*P*-value
Crude	1.04 (0.83–1.29)	0.760	0.84 (0.65–1.08)	0.186	1.30 (0.99–1.70)	0.057
Model 1	1.28 (1.01–1.61)	0.035	1.05 (0.81–1.35)	0.720	1.57 (1.19–2.08)	0.002
Model 2	1.38 (1.06–1.83)	0.016	1.17 (0.87–1.55)	0.294	1.62 (1.18–2.24)	0.003
Model 3	1.38 (1.05–1.80)	0.021	1.16 (0.86–1.56)	0.320	1.67 (1.20–2.31)	0.002
Model 4	1.37 (1.02–1.84)	0.034	1.18 (0.86–1.61)	0.304	1.66 (1.17–2.36)	0.005

*HT* Hypertension, *OR* Odds ratio

Morning HT, meeting home HT criteria (home HT on home BP measurement or under treatment for HT at the secondary survey) in the morning measurement; evening HT, meeting home HT criteria in the evening measurement; and home HT, either or both morning or evening HT

Model 1, age and sex. Model 2, Model 1 plus research heart rate, body mass index, dyslipidemia, diabetes mellitus, estimated glomerular filtration rate, smoking status, alcohol consumption, urinary Na/K, Athens Insomnia Scale score, and regular exercise. Model 3, Model 2 plus educational level, house damage, and examination in winter. Model 4, Model 3 plus average systolic blood pressure (SBP) in the analysis of home HT, morning SBP in the analysis of morning HT, or evening SBP in the analysis of evening HT

Table 4 lists the subgroup analysis for age, sex, BP pattern, and drinking habit. For women participants and non-drinkers, the ORs of depressive symptoms in home and evening HT were significantly high. In those with normotension, depressive symptoms were related to evening HT. In participants with normotension, the OR for hypertension based on research BP was 1.47 (95% CI: 0.99–2.14). Interaction effect with home HT was not observed in any subgroup.

**Table 4:** Odds ratios and 95% CIs for home hypertension at the secondary survey in participants with depressive symptoms according to age, sex, BP patterns, and drinking habit at the baseline survey

	Home HT		Morning HT		Evening HT	
	OR (95% CI)	p-value for interaction	OR (95% CI)	p-value for interaction	OR (95% CI)	*p*-value for interaction
Age (years)						
≤56	1.41 (0.91–2.19)	0.439	1.30 (0.80–2.09)	0.658	1.62 (0.95–2.73)	0.317
>56	1.30 (0.86–1.96)		1.03 (0.66–1.60)		1.48 (0.89–2.42)	
Sex						
Male	1.29 (0.63–2.58)	0.804	1.30 (0.61–2.67)	0.733	2.04 (0.86–4.65)	0.694
Female	1.40 (1.01–1.95)*		1.13 (0.79–1.62)		1.59 (1.07–2.35)*	
BP pattern						
Normotension	1.32 (0.94–1.83)	0.362	1.08 (0.74–1.55)	0.095	1.90 (1.26–2.85)**	0.257
WCHT	1.71 (0.85–3.48)		1.62 (0.81–3.28)		1.22 (0.55–2.68)	
Drinking						
Non-drinker	1.24 (0.82–1.89)	0.942	1.18 (0.74–1.86)	0.411	1.82 (1.11–2.97)*	0.500
Drinker	1.49 (0.98–2.27)		1.17 (0.75–1.81)		1.58 (0.95–2.58)	

*BP* Blood pressure, *HT* Hypertension, *OR* Odds ratio, *ref*. reference, WCHT White-coat hypertension. *P*-value represents p for the interaction. *P-value < 0.05, **P-value < 0.01

Participants are divided according to age, sex, BP patterns, and drinking habit at the baseline survey

Model 4 (age, sex, research heart rate, body mass index, dyslipidemia, diabetes mellitus, estimated glomerular filtration rate, smoking status, alcohol consumption, urinary Na/K, Athens Insomnia Scale, regular exercise, educational level, house damage, examination in winter, and average systolic blood pressure (SBP) in the analysis of home HT, morning SBP in the analysis of morning HT, or evening SBP in the analysis of evening HT) is used for the analysis. Sex in subgroup analysis of sex and alcohol consumption in subgroup analysis of drinking habit were removed from the model

In the sensitivity analysis excluding participants with treated HT at the secondary survey, an elevated OR of depressive symptoms was observed for evening HT (OR: 1.47, 95% CI 0.99–2.15) but not for home HT and morning HT (Supplemental Table S3). The results were similar to those of the main analysis.

## Discussion

This study demonstrated that participants with depressive symptoms had a high risk of home HT, particularly evening HT. Home BP was significantly higher in participants with depressive symptoms than in those without depressive symptoms, whereas research BP, after adjusting for age and sex, did not differ significantly between the groups. The results demonstrated the importance of home BP monitoring.

Previous studies on office BP in the diagnosis of HT have reported debatable results on the association between depressive symptoms and HT. A large-scale prospective cohort study reported that participants with high CES-D scores developed new-onset HT in a median follow-up of 3.6 years [14]. A Taiwanese study, with a few years of follow-up, and a Canadian study, with a 10-year follow-up, reported similar results [13, 15]. Our results were consistent with those of the aforementioned studies; however, other studies reported that depression was related to hypotension [16–19].

There are several possible reasons for the inconsistency in the results of previous studies. First, the relationship between HT and depression may be bidirectional; a prospective cohort study reported that depressive symptoms increased the onset of HT and that those with HT were less likely to be depressed [14]. In cross-sectional studies, due to the coexistence of bidirectional relationships, the associations may be difficult to discern. Notably, most studies reporting negative associations are cross-sectional studies [17–19]. Second, most studies evaluated office BP on one occasion without considering variability and differences between conditions [13–19]. High variability in BP has been reported in individuals with depressive symptoms [40]. Our study is novel in that we evaluated both research and home BP at baseline and secondary surveys. We used the average values of home BP measurement for 10–14 days, and they were less affected by daily BP variability. The secondary survey revealed that home BP was higher in participants with depressive symptoms than in those without depressive symptoms. The results clarified the difference between research and home BP in participants with depressive symptoms, supporting our hypothesis.

Individuals with depression have risk factors for HT, such as DM, obesity, smoking, and inactivity [5]. In our study, the prevalence of DM and dyslipidemia was rather low in those with depressive symptoms, and this may be due to the differences in age and sex between the two groups. Similarly, research BP and home BP were lower in those with depressive symptoms than in those without depressive symptoms, and there was no significant difference after adjusting for age and sex. Our detailed analysis of the relationship between depressive symptoms and home HT using 4 models including these risk factors, revealed that depressive symptoms were an independent risk factor for home HT.

A possible mechanism underlying this relationship is the ANS function. The ANS regulates BP and HR, and its dysregulation results in elevated BP variability, elevated at-rest HR, and reduced HR variability [6]. Sympathetic activity is excessive and parasympathetic activity is inadequate in patients with depression which may explain the elevated home BP [6, 41]. In our study, participants with depressive symptoms had elevated HR, suggesting sympathetic hyperactivity. However, HR variability and autonomic cardiovascular reflex are used to evaluate ANS function in general [42], and it was difficult to evaluate the sympathetic and parasympathetic activities in this study.

Another mechanism may be related to cortisol levels [43]. Mental stress affects the hypothalamic–pituitary–adrenal axis and increases serum cortisol levels [1]. Cortisol levels are reportedly elevated in patients with depression and as depressive symptoms become severe, cortisol levels increase [7, 44]. Cortisol-induced HT is a complex phenomenon involving multiple mechanisms such as sodium retention and volume expansion [45].

In our study, depressive symptoms were related to evening HT but not to morning HT, although both morning and evening BP were higher in participants with depressive symptoms. Japanese lifestyle including drinking alcohol and taking a bath in the evening may affect evening BP. After adjusting alcohol intake and classifying drinking habits, the relationship between depressive symptoms and evening HT was still observed. The mechanism underlying the difference between evening and morning HT in association with depressive symptoms remains unclear. We speculate that diurnal variations in cortisol levels may be attributable to this difference. Serum cortisol levels in healthy individuals are high in the morning and low in the evening [45]. In contrast, a previous study found that cortisol levels in patients with depression were elevated throughout the day, and circadian variation was blunted [44]. Nevertheless, further studies assessing the diurnal variation in cortisol levels are needed to clarify the mechanism.

This study had several limitations. First, individuals with severe depression were not included in this study because even such participants with depressive symptoms could come to the Community Support Center to undergo examinations and measure home BP regularly. In addition, we did not consider the use of anti-depressants, although some anti-depressants reportedly affect BP [46]. Therefore, it is unclear whether these results can be applied to individuals with severe depression. Second, we used the CES-D score to assess depressive symptoms, and its sensitivity for major depressive disorder was reportedly 95.1% [32]. Thus, some individuals with major depressive disorder may have been classified as having non-depressive symptoms. However, their number was estimated to be sufficiently small; therefore, the results were consistent. Third, we did not consider the change in depressive symptoms, because it makes it difficult to distinguish longitudinal and cross-sectional effects on home HT. Depressive symptoms change over time, and the change in depressive symptoms may affect home HT. Lastly, most participants experienced the Great East Japan Earthquake which may have influenced their mental status and management of HT [26, 27]. We adjusted for the extent of house damage level and examination year to reduce these influences. We assumed that the influence was not large enough to affect the results.

## Perspective of Asia

Approximately 150 million people is estimated to have depression in Asia in 2015 [47], and the number is increasing. Adequate approach for mental and somatic problems in individuals with depression is more and more important. As shown in our findings, depressive symptoms even without diagnosis of depression increased the risk of new-onset home HT. This study suggested the importance of monitoring home BP in individuals with depressive symptoms for early diagnosis and management of HT. It may contribute to improving prognosis of many people with depressive symptoms in Asia.

## Conclusion

In conclusion, this study revealed that depressive symptoms are independently associated with new-onset home HT in participants with home normotension. Depressive symptoms should be managed for maintaining mental health and for the prevention of HT and concomitant CVD. Home BP was significantly higher in individuals with depressive symptoms than in those without depressive symptoms, whereas research BP was not. Individuals with depressive symptoms should monitor their home BP for early diagnosis and management of HT, even though they have normotension at clinics and research centers.

## Supplementary Information

Supplementary Information
