# Supplementary material for: Depressive symptoms as risk factors for the onset of home hypertension: a prospective cohort study
Source: Hypertens Res. 2024 Jul 10;47(11):2989–3000. doi: 10.1038/s41440-024-01790-9 (PMC11534705; doi:10.1038/s41440-024-01790-9)
Supplement: Supplementary file 1 — Supplementary Information [file 41440_2024_1790_MOESM1_ESM.docx]

**Supplementary Table 1. Age-and sex–adjusted least squares means and 95% CIs of blood pressure and heart rate at the baseline survey**

|  | Non-depressive symptoms n=2 353 | Depressive symptoms n=729 | *P*-value |
| --- | --- | --- | --- |
| Research SBP (mmHg) | 121.5 (120.9–122.0) | 121.2 (120.3–122.0) | 0.55 |
| Research DBP (mmHg) | 75.2 (74.8–75.7) | 75.1 (74.4–75.9) | 0.80 |
| Research HR (/min) | 65.4 (64.6–65.5) | 65.4 (64.6–66.1) | 0.38 |
| Morning SBP (mmHg) | 118.0 (117.5–118.4) | 118.5 (117.7–119.2) | 0.19 |
| Morning DBP (mmHg) | 71.0 (70.7–71.3) | 71.2 (70.6–71.7) | 0.51 |
| Morning HR (/min) | 66.7 (66.3–67.0) | 67.7 (67.1–68.3) | 0.02 |
| Evening SBP (mmHg) | 115.5 (115.0–115.9) | 115.9 (115.1–116.6) | 0.35 |
| Evening DBP (mmHg) | 67.5 (67.2–67.9) | 67.9 (67.3–68.4) | 0.29 |
| Evening HR (/min) | 69.3 (68.9–69.6) | 69.8 (69.2–70.4) | 0.11 |
| Average home SBP (mmHg) | 116.7 (116.3–117.1) | 117.2 (116.5–117.9) | 0.23 |
| Average home DBP (mmHg) | 69.3 (68.9–69.6) | 69.5 (69.0–70.1) | 0.37 |

DBP, diastolic blood pressure; HR, heart rate; SBP, systolic blood pressure. Average home SBP and DBP were defined as the average of morning and evening SBP and DPB, respectively.

**Supplementary Table 2. Blood pressure, heart rate, and health-related characteristics at the secondary survey 2017– 2020**

| Depressive symptoms at the baseline survey | Non-depressive symptoms n=2 353 | Depressive symptoms n=729 | *P* value |
| --- | --- | --- | --- |
| **Blood pressure and heart rate, mean±SD** | |  |  |
| Research SBP (mmHg) | 120.4±15.4 | 118.0±15.2 | <0.001 |
| Research DBP (mmHg) | 74.0±9.6 | 73.7±9.7 | 0.446 |
| Research HR (/min) | 66.2±9.2 | 67.0±9.0 | 0.041 |
| Number of morning BP measurements | 9.4±1.8 | 9.2±2.2 | 0.005 |
| Morning SBP (mmHg) | 118.4±13.0 | 117.7±13.1 | 0.181 |
| Morning DBP (mmHg) | 71.1±8.5 | 71.2±8.7 | 0.808 |
| Morning HR (/min) | 66.3±7.6 | 67.8±8.1 | <0.001 |
| Number of evening BP measurements | 9.6±2.0 | 9.4±2.3 | 0.001 |
| Evening SBP (mmHg) | 115.2±12.7 | 115.0±13.3 | 0.827 |
| Evening DBP (mmHg) | 67.5±8.2 | 68.3±9.2 | 0.025 |
| Evening HR (/min) | 68.6±7.7 | 69.3±8.1 | 0.038 |
| Average home SBP (mmHg) | 116.8±12.2 | 116.4±12.5 | 0.404 |
| Average home DBP (mmHg) | 69.3±8.0 | 69.8±8.5 | 0.205 |
| **Health-related characteristics** | | |  |
| BMI, mean±SD | 21.9±3.0 | 21.8±3.3 | 0.353 |
| HbA1c (%), mean±SD | 5.53±0.40 | 5.52±0.42 | 0.572 |
| LDL (mg/dL), mean±SD | 123.9±30.0 | 123.7±29.2 | 0.930 |
| HDL (mg/dL), mean±SD | 66.9±16.7 | 68.3±17.4 | 0.083 |
| TG (mg/dL), mean±SD | 88.7±54.2 | 88.6±57.3 | 0.961 |
| G-GTP (IU), mean±SD | 24.9±24.5 | 23.4±19.7 | 0.156 |
| eGFR (mL/min/1.73 m^2^), mean±SD | 77.0±13.1 | 78.1±13.4 | 0.090 |
| Urine Na/K ratio, mean±SD | 4.3±0.87 | 4.3±0.88 | 0.727 |
| Alcohol consumption (g/day), median (IQR) | 0.00 (0.00–9.43) | 0.00 (0.00–4.27) | <0.001 |
| Smoking status, n (%) |  |  | 0.792 |
| Never smoker | 1638 (69.6) | 446 (61.2) |  |
| Former smoker | 487 (22.8) | 23.4 (15.5) |  |
| Current smoker | 117 (5.0) | 31 (4.3) |  |
| Regular exercise, n (%) | 1914 (89.0) | 505 (82.4) | <0.001 |
| CES-D | 9.3±5.8 | 18.0±8.5 | <0.001 |
| Depressive symptoms | 257 (10.9) | 437 (59.9) | <0.001 |

BMI, body mass index; BP, blood pressure; CES-D, Center for Epidemiologic Studies Depression Scale; DBP, diastolic blood pressure; eGFR, estimated glomerular filtration rate; G-GTP, gamma-glutamyl transpeptidase; HbA1c, hemoglobin A1c; HDL, high-density lipoprotein; HR, heart rate; LDL, low-density lipoprotein; SBP, systolic blood pressure; TC, total cholesterol; TG, triglyceride.

**Supplementary Table 3. Age- and sex-adjusted least square means of blood pressure and change in blood pressure between the baseline and the secondary surveys, and the prevalence of home hypertension at the secondary survey 2017– 2020 excluding participants under treatment for hypertension**

|  | Non-depressive symptoms n=2 353 | Depressive symptoms n=729 | *P*-value |
| --- | --- | --- | --- |
| **Blood pressure and heart rate**, age- and sex-adjusted least square means (95%CI) | | | |
| Research SBP (mmHg) | 121.0 (120.0–122.0) | 121.0 (120.0–122.0) | 0.95 |
| Research DBP (mmHg) | 74.9 (74.4–75.3) | 74.8 (74.1–75.6) | 0.94 |
| Research HR (/min) | 119.0 (118.7–119.8) | 120.5 (119.5–121.5) | 0.026 |
| Morning SBP (mmHg) | 72.1 (71.7–72.5) | 72.7 (72.0–73.4) | 0.102 |
| Morning DBP (mmHg) | 116.0 (115.0–117.0) | 117.0 (116.0–118.0) | 0.017 |
| Morning HR (/min) | 68.1 (67.7–68.5) | 69.1 (68.4–69.8) | 0.009 |
| Evening SBP (mmHg) | 117.6 (117.0–118.1) | 118.8 (117.9–119.8) | 0.015 |
| Evening DBP (mmHg) | 70.1 (69.7–70.5) | 70.9 (70.2–71.6) | 0.027 |
| **Change in blood pressure between the baseline and secondary surveys**, age- and sex-adjusted least square means (95% CI) | | | |
| Research SBP (mmHg) | -0.7 (-1.3–-0.1) | -0.1 (-1.0–0.9) | 0.215 |
| Research DBP (mmHg) | -0.3 (-0.7–0.03) | -0.1 (-0.7–0.5) | 0.508 |
| Morning SBP (mmHg) | 1.3 (0.8–1.7) | 2.0 (1.3–2.8) | 0.063 |
| Morning DBP (mmHg) | 1.2 (0.9–1.5) | 1.6 (1.1–2.1) | 0.105 |
| Evening SBP (mmHg) | 0.6 (0.2–1.1) | 1.6 (0.8–2.4) | 0.017 |
| Evening DBP (mmHg) | 0.6 (0.3–1.0) | 1.3 (0.8–1.9) | 0.015 |

DBP, diastolic blood pressure; HR, heart rate; SBP, systolic blood pressure. Average home SBP and DBP were defined as the average of morning and evening SBP and DPB, respectively.

**Supplementary Table 4. The prevalence, odds ratios, and 95% CIs for home hypertension in participants with depressive symptoms excluding participants under treatment for hypertension at the secondary survey.**

|  | Prevalence in non-depressive symptoms  n=2 297  n (%) | Prevalence in depressive symptoms  n= 711  n (%) | OR (95%CI) | *P*-value |
| --- | --- | --- | --- | --- |
| Home HT | 346 (15.2) | 108 (15.3) | 1.26 (0.92–1.71) | 0.139 |
| Morning HT | 305 (13.3) | 78 (11.0) | 1.07 (0.76–1.50) | 0.673 |
| Evening HT | 163 (7.1) | 65 (9.2) | 1.47 (0.99–2.15) | 0.051 |

HT, hypertension; OR, odds ratio.

Treated hypertension was defined as being under treatment for HT at the secondary survey, according to the questionnaire.

Morning HT, meeting home HT criteria (home HT on home BP measurement) in the morning measurement; evening HT, meeting home HT criteria in the evening measurement; and home HT, either or both morning or evening HT.

Model 4 including age, sex, research heart rate, body mass index, dyslipidemia, diabetes mellitus, estimated glomerular filtration rate, smoking status, alcohol consumption, urinary Na/K, Athens Insomnia Scale score, regular exercise, educational level, house damage, examination in winter, and average home systolic blood pressure (SBP) in analysis for home HT, morning SBP in the analysis for morning HT or evening SBP in the analysis for evening HT.
